# Aristolochic acid I accelerates lung adenocarcinoma progression coupled with the upregulation of core oncogenic networks: an integrated network toxicology and experimental study

**DOI:** 10.3389/fphar.2026.1860873

**Published:** 2026-07-15

**Authors:** Linchuan Mo, Fenglei Yu, Muyun Peng, Yu He, Shouzhi Xie, Shengrong Wu, Zhe Zhang, Cheng Wang, Wangcheng Zhao, Yifan Ouyang, Xuyang Yi, Li Wang, Xinge Peng, Xinchun Huang, Qikang Hu, Juan Luo, Xiaofeng Chen, Zhi Yang

**Affiliations:** 1 Department of Anesthesiology, Second Xiangya Hospital, Central South University, Changsha, China; 2 Department of Thoracic Surgery, The Second Xiangya Hospital, Central South University, Changsha, China; 3 Hunan Key Laboratory of Early Diagnosis and Precision Treatment of Lung Cancer, The Second Xiangya Hospital of Central South University, Changsha, China; 4 Department of Thoracic and Cardiovascular Surgery, The First Affiliated Hospital, Hengyang Medical School, University of South China, Hengyang, Hunan, China

**Keywords:** aristolochic acid I, experimental validation, LUAD, molecular docking, phytotoxin, prognostic analysis

## Abstract

**Background:**

Aristolochic Acid I (AAI) is a potent nephrotoxin and Group 1 carcinogen. Despite stringent regulatory restrictions, AAI-containing herbal remedies are still sporadically used to treat respiratory symptoms, presenting a previously underappreciated exposure risk for patients with lung adenocarcinoma (LUAD). However, the specific tumor-promoting effects of AAI on preexisting LUAD remain to be fully elucidated.

**Methods:**

In this study, we integrated network toxicology, TCGA transcriptomic analysis, and molecular docking to identify core oncogenic networks potentially affected by AAI. The associated pro-tumor phenotypes and transcriptional regulatory abnormalities were subsequently investigated through a series of *in vitro* and *in vivo* experiments.

**Results:**

Network analysis and TCGA data mining identified a cluster of seven hub genes—including ERBB2, SERPINE1, CCNA2, and CHEK1—that are crucial to LUAD progression and significantly associated with poor clinical prognosis. Molecular docking simulations suggested potential binding affinities between AAI and these target proteins. Functional assays demonstrated that acute AAI exposure significantly accelerated the proliferation, migration, and invasion of LUAD cell lines (PC9 and NCI-H1299) *in vitro*, while promoting macroscopic xenograft tumor growth *in vivo*. The RT-qPCR and WB results showed that AAI exposure was accompanied by an increase in the expression of core genes.

**Conclusion:**

Our findings suggest that, beyond its established chronic mutagenic toxicity, AAI may act as a potent tumor promoter in LUAD. By potentially influencing key oncogenic networks, AAI accelerates the malignant progression of LUAD, underscoring the severe clinical hazards of AAI exposure in patients with preexisting lung malignancies.

## 1 Introduction

Lung cancer is one of the most common cancers in the world (Brody, 2020; Nasim et al., 2019). Lung adenocarcinoma (LUAD) is the most common histological subtype of non-small-cell lung cancer (Mogavero et al., 2023). Despite significant advances in diagnostic methods (such as high-resolution CT) and treatments (including targeted therapy and immunotherapy), the prognosis for patients with LUAD remains poor owing to rapid tumor progression and a high rate of acquired resistance (Li et al., 2024; Yanagawa et al., 2021). Prevention and early intervention have become new directions in LUAD research (Nooreldeen and Bach, 2021; Slatore and Sockrider, 2014). Smoking, stress, genetic factors, environmental carcinogens, and emotional factors are common risk factors associated with LUAD (Bade and Dela Cruz, 2020). Reducing and avoiding exposure to a range of carcinogenic factors are key to preventing LUAD.

Aristolochic Acids (AA) are a group of nitrophenanthrene carboxylic acids that exist in plants in the Aristolochiaceae family and Asarum species (Zhang et al., 2022). These are significant components of Chinese herbal medicines with documented use. Chinese herbs containing AAI, such as Aristolochia and Guanmutong, have been used to treat diseases, such as rheumatoid arthritis, pneumonia, and stroke (Yang et al., 2018). Until the 1990s, over 100 Belgian women were diagnosed with aristolochic acid nephropathy (AAN), and the toxicity of AA began to attract attention; extensive research has been conducted. There is substantial evidence of nephrotoxicity, hepatotoxicity, and carcinogenicity of AA (Dickman et al., 2023; Das et al., 2022; Chin et al., 2024). In 2012, the International Agency for Research on Cancer (IARC) classified AA as a Group 1 human carcinogen. Since 2003, the Chinese Pharmacopoeia has sequentially removed four AA-containing herbal medicines. However, in 2017, the China Food and Drug Administration (CFDA) reported that more than 40 Chinese patent medicines and 24 herbal medicines containing AA are still in clinical use (Zhang et al., 2022).

Therefore, we may ingest AA or medications containing AA. Aristolochic Acid I (AAI) and Aristolochic Acid II (AAII) are representative components of AA compounds with the highest content and the strongest toxicity, especially AAI (Kwok et al., 2025). Currently, there is no in-depth research on the effects of AAI on LUAD. Owing to its therapeutic effects on respiratory diseases, patients with LUAD are likely to take aristolochic acid-containing medications for respiratory symptoms. At the same time, considering the carcinogenicity of AAI, it is particularly important to explore its effects on LUAD. This study aimed to elucidate the potential tumor-promoting effects and associated molecular networks of AAI in LUAD, thereby providing vital scientific evidence for stringent clinical drug safety and prevention of accelerated tumor progression in high-risk populations.

The workflow diagram outlines the process from network toxicology research to experimental validation in investigating the role of AAI in promoting lung adenocarcinoma progression.

## 2 Materials and methods

### 2.1 Toxicity identification and target screening of AAI

The SMILES string and chemical structure of Aristolochic Acid I (AAI) were retrieved from PubChem (https://pubchem.ncbi.nlm.nih.gov). Compound toxicity was predicted and evaluated using ADMETlab 2.0 and ProTox 3.0. Potential drug targets of AAI were identified by searching three databases: SwissTargetPrediction (https://swisstargetprediction.ch/), ChEMBL (https://www.ebi.ac.uk/chembl/), and the Similarity Ensemble Approach (SEA). Duplicate targets were removed using the UniProt database to establish the final AAI target set.

### 2.2 Acquisition of targets for LUAD

GeneCards (https://www.genecards.org/) and OMIM (https://www.omim.org/) databases were used to search for gene targets related to LUAD, then merging the results, removing duplicates, and standardizing the data. The search term is “lung adenocarcinoma,” and the species is *Homo sapiens*.

### 2.3 Construction of the protein-protein interaction network and screening of core targets

The intersecting targets of AAI and LUAD were imported into the STRING platform (species: *H. sapiens*, minimum confidence: 0.7) to construct a protein-protein interaction network. After removing isolated nodes, the network was imported into Cytoscape 3.10.3 to analyze each gene’s Betweenness Centrality, Closeness Centrality, Degree, Radiality, Stress, and MCC values (Wang et al., 2025). Genes with all indicators above the median were selected as the core targets.

### 2.4 Analysis of potential target gene functions and pathway enrichment

The intersecting targets of AAI and LUAD were analyzed for Gene Ontology (GO) and Kyoto Encyclopedia of Genes and Genomes (KEGG) enrichment using the ‘org. Hs.eg.db’ and ‘clusterProfiler’ packages. The analysis results were visualized using the ‘enrichplot’ package.


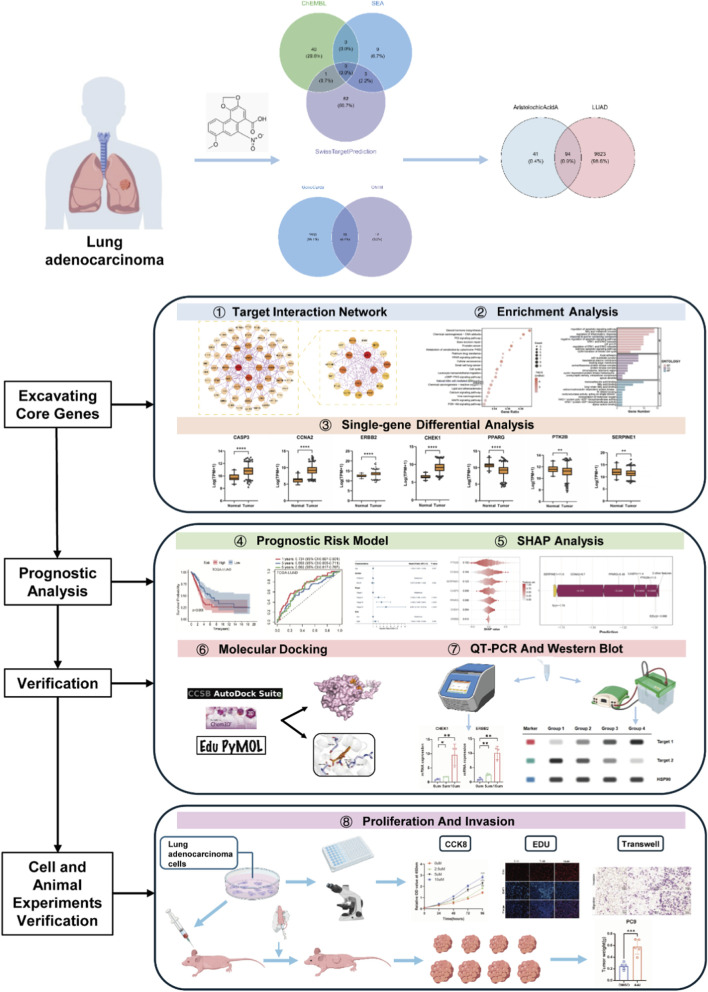


### 2.5 TCGA transcriptome data analysis and core hub gene screening

Transcriptome data were obtained from the TCGA database, and differential analysis was conducted using the ‘DEG’ package. Single-gene expression analysis was performed on the intersection of differentially expressed genes and core genes, which are the core hub genes. Further analysis and visualization of the dataset were carried out in GraphPad Prism 10.1.2.

### 2.6 Molecular docking of AAI with core hub targets

The 3D structures of AAI and the core hub proteins were obtained from PubChem and UniProt, respectively. Both ligands and receptors were preprocessed using PyMOL and then imported into AutoDockTools for molecular docking. The binding poses and interactions were visualized with PyMOL.

### 2.7 External validation of the GEO database

To externally validate the expression profiles and prognostic value of the identified core hub genes, four independent microarray datasets (GSE31210, GSE43458, GSE81089, and GSE68465) were downloaded from the Gene Expression Omnibus (GEO) database (https://www.ncbi.nlm.nih.gov/geo/).

Using the GSE31210, GSE43458, and GSE81089 datasets, the mRNA expression levels of the core genes were analyzed and compared between LUAD tissues and normal control tissues.

Using GSE31210, GSE68465, and GSE81089 as external validation cohorts, patients in these GEO cohorts were divided into high-risk and low-risk groups using the same risk score formula derived from the TCGA dataset. Kaplan-Meier survival curves were plotted to compare overall survival (OS) between the two groups. In addition, time-dependent receiver operating characteristic (ROC) curves were generated to evaluate the predictive accuracy of the risk model. Finally, univariate and multivariate Cox regression analyses were performed to confirm whether the risk model could serve as an independent prognostic indicator for LUAD patients.

### 2.8 Establishment of prognostic risk model and SHAP analysis

Cox regression analysis was performed using gene expression and clinical data from the TCGA dataset to construct a prognostic risk model. The “survminer” package was used to determine the optimal cutoff point for stratifying patients into high-risk and low-risk groups. Kaplan-Meier curves were generated to compare overall survival (OS). SHAP analysis was applied to evaluate the individual contribution of each core hub gene to the prediction model.

### 2.9 CCK-8 assay

In the CCK-8 assay, NCI-H1299 and PC9 cells were harvested by trypsin digestion, and after counting, 1,000 cells were seeded per well in a 96-well plate. The cells were treated with different concentrations of AAI at 2.5 μM, 5 μM, 10 μM, 25 μM, 50 μM, 100 μM, and 500 μM (Li Y. et al., 2023; Liu et al., 2022; Xie et al., 2024). Control cells were exposed to an equal volume of dimethyl sulfoxide (DMSO). At five time points after treatment—0 h, 24 h, 48 h, 72 h, and 96 h—cell viability was measured by absorbance at 450 nm using the CCK-8 assay kit (GLPBIO, Cat. No. GK1001) following the manufacturer’s instructions.

### 2.10 EdU cell proliferation assay

NCI-H1299 and PC9 cells were seeded into 96-well plates at a density of 10,000 cells per well and cultured in RPMI-1640 medium supplemented with 10% serum and 5 μM or 10 μM AAI for 24 h. Cell proliferation was then assessed using the BeyoClick™ EdU-594 Cell Proliferation Detection Kit (Beyotime Biotechnology) according to the manufacturer’s instructions. Fluorescent images were recorded using a fluorescence microscope (TH4-200, Olympus).

### 2.11 Wound healing assay

Cells were seeded in 10-cm dishes and then distributed into 6-well plates to achieve 70%–80% confluence. A linear scratch wound was created by gently scraping the cell monolayer twice with a sterile pipette tip, followed by two washes with PBS. The cells were subsequently cultured in serum-free medium for 30 h. Images of the wound area were recorded using a microscope (model TH4200, Olympus).

### 2.12 Cell invasion and migration assay

To evaluate the effect of AAI on cell migration and invasion capabilities, we conducted experiments using Transwell chambers (pore size 8 μm; BIOFIL). NCI-H1299 or PC9 cells were first pretreated for 24 h in serum-free RPMI-1640 medium containing 5 μM or 10 μM AAI, and then 3 × 10^4^ cells were seeded in the upper chamber of the Transwell (serum-free medium). In the migration assay, the lower chamber was filled with RPMI-1640 medium containing 10% serum and 5 μM or 10 μM AAI as a chemoattractant. After 48 h of incubation, the membrane was fixed with 4% paraformaldehyde for 20 min, stained with crystal violet, and the cells that had migrated through the membrane were observed and counted directly under a microscope (BX53, Olympus). In the invasion assay, the Transwell chambers were pre-coated with Matrigel™ matrix (#354234, Corning, United States) according to the manufacturer’s instructions. The lower chamber contained RPMI-1640 medium with 20% serum and 5 μM or 10 μM AAI, and the chambers were incubated for 30 h. Non-invaded cells on the upper surface of the membrane were removed, then the membrane was fixed and stained, and the number of invaded cells per field was quantified using an inverted microscope imaging system (BX53, Olympus).

### 2.13 RNA extraction and quantitative RT-qPCR analysis

Total RNA was extracted using the RNAfast200 purification kit (Feijie, Shanghai) following the manufacturer’s instructions. cDNA was synthesized from the RNA using the ReverTra Ace RT PCR kit (TOYOBO Biotechnology, Japan). All RT PCR reactions were performed with SYBR Green Master Mix (Roche Molecular Systems, Switzerland). Β-Actin was used as the internal control. Primer sequences are shown in Supplementary Table S1.

### 2.14 Western blot

PC9 and NCI-H1299 cells treated with AAI (10 μM) or DMSO were harvested, and total protein was extracted using RIPA lysis buffer (Solarbio, China) supplemented with phenylmethylsulfonyl fluoride (PMSF) and a protease inhibitor cocktail (MedChem-Express, China). Protein concentrations were quantified using a BCA Protein Assay Kit (Solarbio) following the manufacturer’s instructions. SDS-PAGE separated equal amounts of protein samples and subsequently transferred onto nitrocellulose membranes. The membranes were blocked with 3% bovine serum albumin (BSA) at 37 °C for 1 h and then incubated overnight at 4 °C with the following primary antibodies: CCNA2 (#AWA11009, Abiowell, 1:2,000), SERPINE1 (#AWA03888, Abiowell, 1:2000), CHEK1 (#AWA00292, Abiowell, 1:7,000), and HSP90 (#13171-1-AP, Proteintech, 1:10,000). HSP90 was utilized as internal loading control. After washing, membranes were incubated with HRP-conjugated secondary antibodies, including HRP-conjugated Goat Anti-Mouse IgG (#LF101, YaMei) and HRP-conjugated Goat Anti-Rabbit IgG (#LF102, YaMei), at 37 °C for 1 h. Finally, the specific protein bands were visualized using an enhanced chemiluminescence (ECL) substrate (APExBIO, United States).

### 2.15 *In vivo* tumor formation

All animal-related procedures were approved by the Experimental Animal Ethics Committee of the First Affiliated Hospital of the University of South China (Ethical Approval No.: 2023LL1222001). Four-week-old female BALB/c nude mice were purchased from Hunan SJA Laboratory Animal Co., Ltd. (China). All animals were housed in a specific pathogen-free (SPF) facility maintained at 22 °C ± 2 °C with 50% ± 10% humidity, under a 12-h light/dark cycle, and were provided *ad libitum* access to food and water.

To establish the xenograft model, each mouse was subcutaneously inoculated into the right dorsal flank with 6 × 10^6^ PC9 cells suspended in 100 μL of PBS. Following inoculation, the mice were randomly assigned to either the experimental or control group (n = 5 per group). Starting on day 5 post-inoculation, mice in the experimental group received intraperitoneal injections of AAI (4 mg/kg) every other day (Xie et al., 2024; Sun et al., 2023; Liu et al., 2021). The AAI was initially dissolved into DMSO and then diluted with PBS to the working concentration (final DMSO concentration ≤0.1%). Mice in the control group were intraperitoneally administered an equal volume of PBS containing an equivalent concentration of DMSO on the same schedule.

Throughout the study, body weight, general activity, and tumor size were monitored every 3 days. Tumor dimensions were measured using a digital vernier caliper, and tumor volume was calculated using the formula: volume = (length × width^2^)/2. Tumor growth curves were subsequently generated. During the experiment, humane endpoints were established; mice were euthanized early and recorded as deaths if the tumor volume exceeded 2,000 mm^3^, tumor ulceration occurred, body weight decreased by more than 20%, or the animals exhibited a moribund state. All mice were uniformly euthanized on day 50. Euthanasia method: in a dedicated CO2 euthanasia chamber with CO2 concentration increasing from 40% to 100%, replacing 30%–70% of the chamber volume per minute for 5–10 min, until cessation of heartbeat and respiration was confirmed to verify death. The tumors were excised and weighed (in grams).

## 3 Results

### 3.1 Compound toxicity prediction analysis

Toxicity assessments on both ProTox 3.0 and ADMETlab 2.0 platforms indicated that AAI poses an extremely high risk for carcinogenicity and respiratory toxicity (Supplementary Figures S2, S3). These prediction scores align with the established characteristics of Group 1 carcinogens (Table 1).

**TABLE 1:** The predicted toxicity of AAI.

System	ProTox-3.0	ADMETlab2.0	Toxicity
Carcinogenicity	0.77	0.935	(+)
Respiratory toxicity	0.59	0.946	(+)

### 3.2 Target acquisition and intersection genes

**FIGURE 1:**
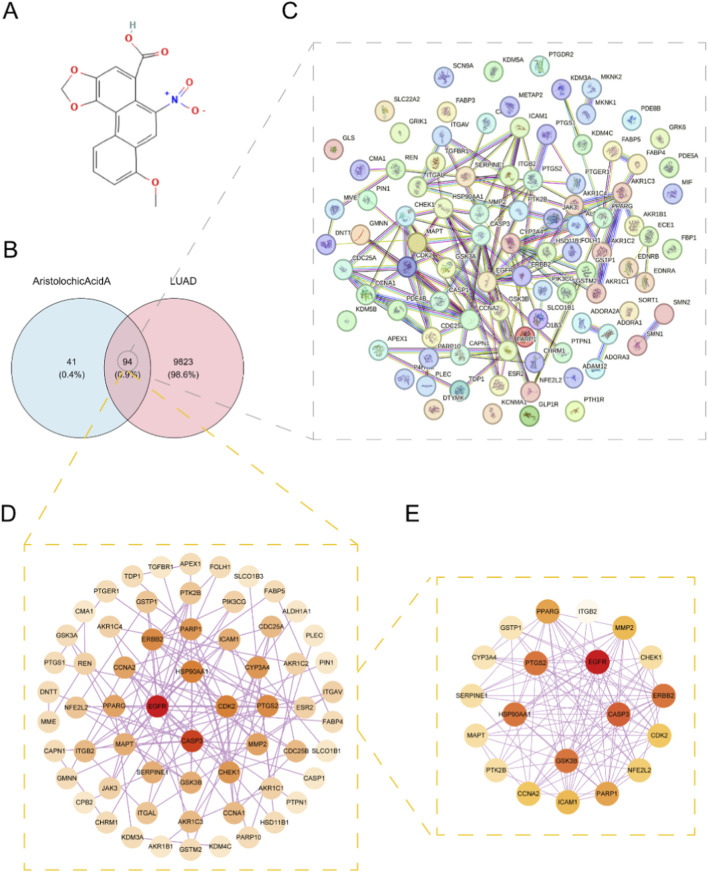
AAI structure and targets affect the progression of lung adenocarcinoma. **(A)** Chemical structure of AAI. **(B)** Intersection of target genes of AAI and lung adenocarcinoma. **(C)** Protein interaction network of 94 targets. **(D)** Protein interaction network of 62 targets. **(E)** Protein interaction network of 20 core targets.

Data integration across multiple databases yielded 135 AAI-related targets and 9,917 lung adenocarcinoma-related genes. Intersection analysis identified 94 overlapping targets between AAI and the disease (Figure 1B). These overlapping genes were considered the candidate set, mediating the regulatory effects of AAI on lung adenocarcinoma (Figure 1A).

### 3.3 Construction of the PPI network and screening of core targets

A PPI network of the 94 intersecting targets was constructed using the STRING database. After removing single-node genes, a sub-network comprising 62 nodes and 124 edges was analyzed in Cytoscape (Figures 1C,D). Based on six topological scoring criteria, 20 core genes with scores above the median were selected for further analysis (Table 2; Figure 1E).

**TABLE 2:** Top 20 core genes by score.

Number	Gene name	Degree	Betweenness centrality	Closeness centrality	Radiality	Stress	MCC score
1	EGFR	16	0.462287503	0.4728682	0.9303279	4,810	38
2	CASP3	14	0.258755823	0.4295775	0.9170082	3,628	49
3	CDK2	10	0.069032565	0.3370166	0.8770492	1,628	146
4	HSP90AA1	10	0.078951869	0.3961039	0.9047131	1,250	35
5	ERBB2	9	0.037159180	0.3812500	0.8985656	526	20
6	PTGS2	9	0.111289942	0.4013158	0.9067623	1,314	22
7	CHEK1	8	0.036614473	0.3315217	0.8739754	1,060	138
8	CYP3A4	8	0.268306011	0.2975610	0.8524590	2,832	8
9	PARP1	8	0.147686255	0.3836478	0.8995902	1,606	13
10	CCNA2	7	0.024805299	0.3177083	0.8657787	672	128
11	MMP2	7	0.052099110	0.3567251	0.8872951	834	15
12	PPARG	7	0.151726949	0.3696970	0.8934426	1,626	8
13	GSK3B	6	0.023064722	0.3567251	0.8872951	382	16
14	ICAM1	6	0.033664952	0.3567251	0.8872951	618	12
15	MAPT	6	0.051455894	0.3652695	0.8913934	452	15
16	SERPINE1	6	0.098718094	0.3193717	0.8668033	1,388	10
17	ITGB2	5	0.019262831	0.3426966	0.8801230	244	10
18	GSTP1	4	0.302186660	0.3719512	0.8944672	3,250	4
19	NFE2L2	4	0.081832139	0.3765432	0.8965164	1,274	4
20	PTK2B	4	0.008120494	0.3315217	0.8739754	166	6

### 3.4 Function and enrichment analysis of targets

**FIGURE 2:**
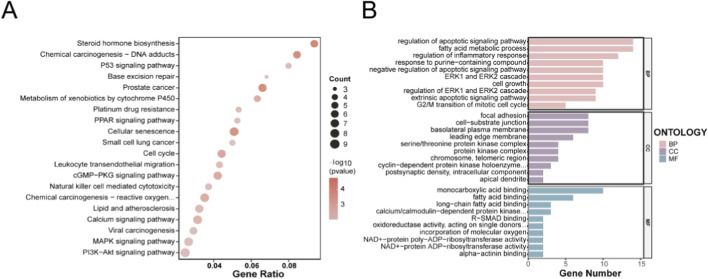
GO and KEGG enrichment analyses were performed on the overlapping targets of AAI and lung adenocarcinoma. **(A)** The gene-count percentages for the top 20 most enriched KEGG signaling pathways were visualized using Gene Ratio. Bubble size represented the number of enriched targets in each pathway, while bubble color intensity reflected the enrichment significance. **(B)** GO analysis was displayed as a histogram, showing the number of genes for the top 10 terms across biological process (BP), cellular component (CC), and molecular function (MF) for the candidate targets.

GO functional enrichment analysis showed that the intersecting targets are mainly involved in biological processes such as regulation of apoptosis signaling, reprogramming of fatty acid metabolism, inflammatory response, and disruption of the ERK signaling pathway. They are localized in core structures that regulate cell adhesion and migration, such as focal adhesions and cell-matrix junctions (Figure 2A). KEGG pathway enrichment revealed that these targets are involved in key carcinogenic pathways, including steroid hormone biosynthesis, chemical carcinogenesis-DNA adduct formation, and the p53 pathway (Figure 2B), suggesting that AAI may drive the development of LUAD through multi-pathway synergy.

### 3.5 TCGA transcriptome validation

**FIGURE 3:**
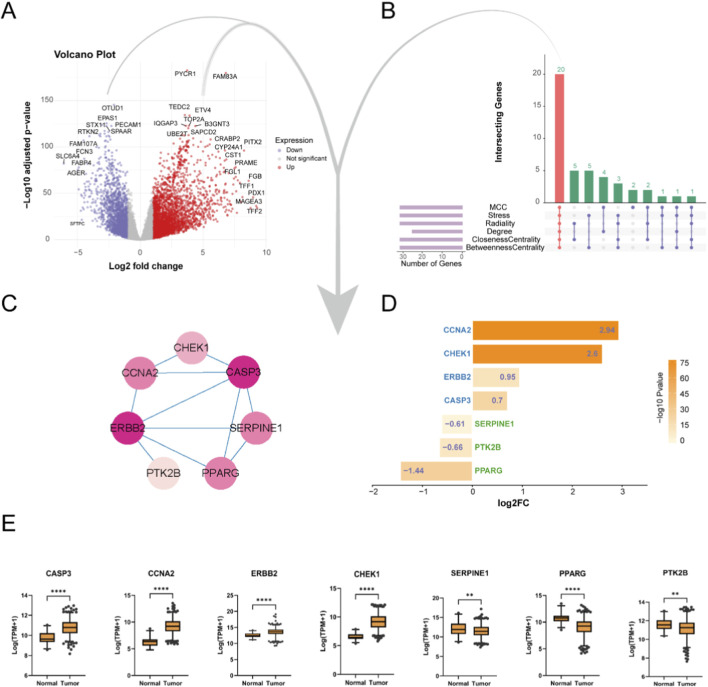
Identification of core hub genes and their differential expression. **(A)** Volcano plot of DEGs in the TCGA lung adenocarcinoma dataset, with red representing significantly upregulated genes and blue representing significantly downregulated genes. **(B)** 20 core targets were selected based on six scoring criteria in Cytoscape 3.10.3. **(C)** 7 core hub genes: PPARG, CASP3, CCNA2, CHEK1, PTK2B, SERPINE1, and ERBB2. **(D)** Upregulation and downregulation relationships of core hub genes. **(E)** Differential expression of core hub genes in normal and lung adenocarcinoma samples. (*p < 0.05, **p < 0.01, ***p < 0.001, ****p < 0.0001).

We obtained 541 LUAD samples and 59 normal control samples from the TCGA database. By analyzing these, we identified significantly differentially expressed genes and validated the differential expression of 20 core genes selected from the protein interaction network, ultimately retaining 7 core hub genes with significant differences: PPARG, CASP3, CCNA2, CHEK1, PTK2B, SERPINE1, and ERBB2 (Figures 3A–D). Subsequently, we performed single-gene differential analysis on these 7 genes, and found that in LUAD patients, CASP3, CCNA2, CHEK1, and ERBB2 were significantly upregulated, while PPARG, PTK2B, and SERPINE1 were significantly downregulated (Figure 3E). At the same time, in GSE31210 and GSE43458, the mRNA expression level of SERPINE1 showed a slight decrease, but it is not statistically significant, whereas in GSE81089, the mRNA expression level of SERPINE1 was significantly increased (Supplementary Figures S1A–C).

### 3.6 Molecular docking validation

**FIGURE 4:**
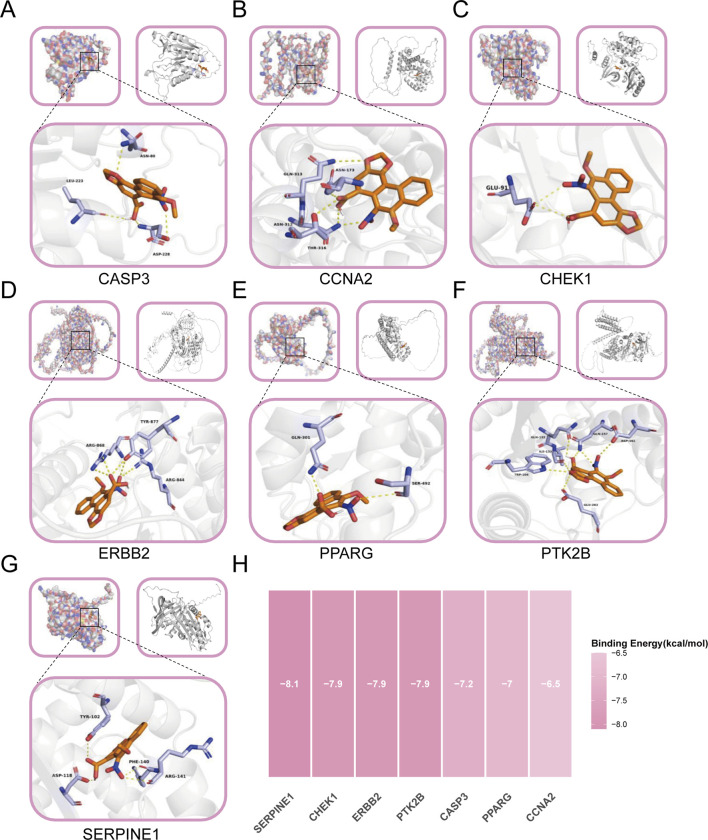
Molecular docking results of AAI with the target protein. **(A–G)** Binding sites of AAI molecules within each core protein structural pocket and their interactions with residues. **(H)** Binding energy between AAI and each core protein.

In silico molecular docking simulations were performed to predict the binding potential between AAI and the seven core hub proteins. Computational analysis suggested that AAI might have high theoretical binding affinities to all seven targets, potentially fitting into their structural pockets with predicted binding energies below −6.0 kcal/mol (Figure 4). Among these predictive models, SERPINE1 and AAI show the highest binding energy of −8.1 kcal/mol (Figure 4H).

### 3.7 Prognostic risk model and SHAP analysis

**FIGURE 5:**
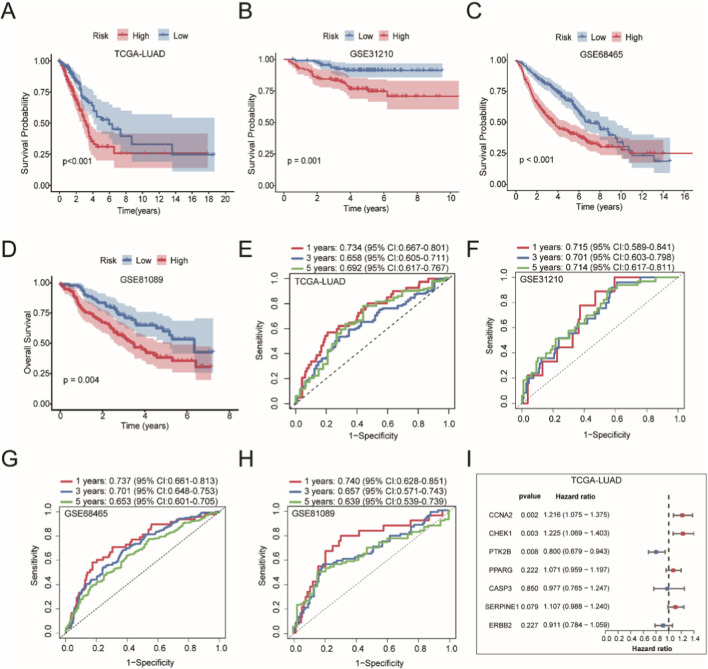
Construction and comprehensive external validation of the core hub gene-based prognostic risk model. **(A)** Kaplan-Meier survival analysis comparing the overall survival (OS) of high-risk and low-risk lung adenocarcinoma patients in the TCGA-LUAD training cohort (p < 0.001). **(B–D)** Kaplan-Meier survival curves demonstrating the robust prognostic stratification of the risk model in three independent external validation cohorts: **(B)** GSE31210, **(C)** GSE68465, and **(D)** GSE81089. **(E)** Time-dependent Receiver Operating Characteristic (ROC) curves demonstrating the predictive accuracy of the risk model in the TCGA-LUAD cohort at 1-, 3-, and 5-year time points. **(F–H)** Time-dependent ROC curves verifying predictive accuracy in the external validation cohorts: **(F)** GSE31210, **(G)** GSE68465, and **(H)** GSE81089, with corresponding 1-, 3-, and 5-year AUCs and 95% Confidence Intervals (CIs) shown. **(I)** Forest plot of the Cox proportional hazards regression analysis identifying the prognostic value of the individual core hub genes (CCNA2, CHEK1, PTK2B, PPARG, CASP3, SERPINE1, and ERBB2) in the TCGA-LUAD dataset.

To evaluate the impact of AAI-related core genes on the prognosis of patients with lung adenocarcinoma (LUAD), we constructed a prognostic risk model and performed independent prognostic analyses. As illustrated in Figure 5, survival analysis of the TCGA training cohort revealed that patients in the high-risk group experienced significantly worse overall survival (OS) compared to those in the low-risk group (Figure 5A). Time-dependent ROC analysis demonstrated that the risk prediction model exhibited acceptable predictive performance at 1, 3, and 5 years (Figure 5E). To address potential overfitting and validate the robustness of the model, we performed external validation using three independent LUAD cohorts from the GEO database (GSE31210, GSE68465, and GSE81089). The Kaplan-Meier curves and ROC analysis indicated that the model maintained consistent prognostic stratification ability across all external datasets (Figures 5B–D, F–H).

**FIGURE 6:**
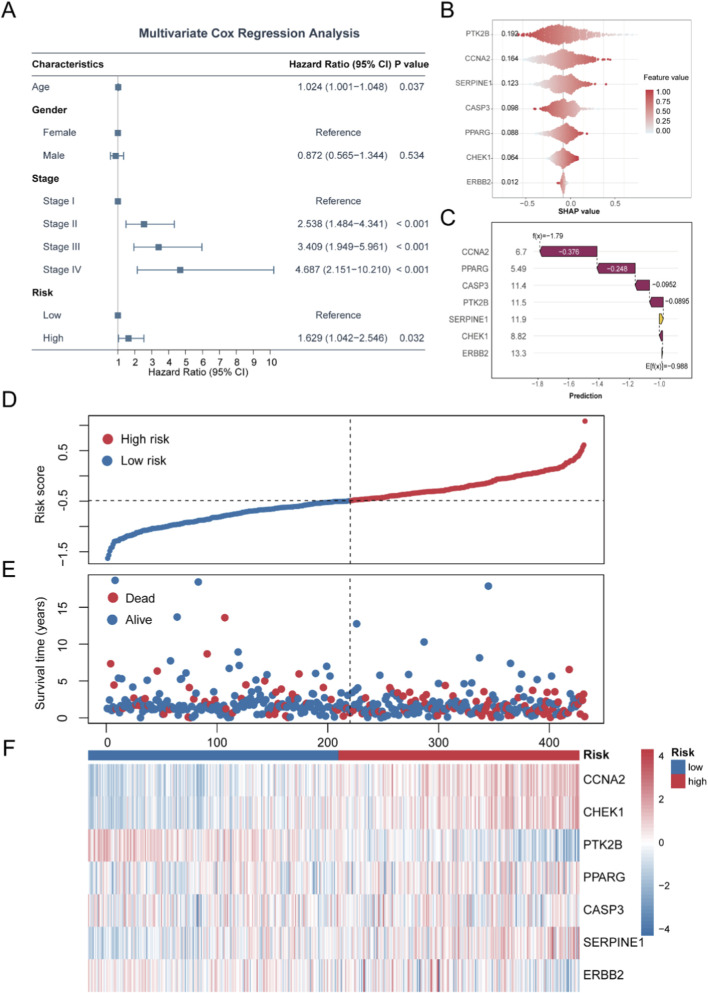
Independent prognostic evaluation of the risk model and SHAP-based interpretability. **(A)** Multivariate Cox regression analysis (forest plot) incorporating clinical characteristics (Age, Gender, Stage) and the Risk score, demonstrating that the High-risk group is an independent prognostic factor associated with poor survival (Hazard Ratio = 1.629, 95% CI: 1.042–2.546, p = 0.032). **(B)** SHAP summary plot, ranking the global importance of core hub genes in the risk model output. **(C)** SHAP waterfall chart, showing the local explanation for a specific sample and the individualized contributions of each gene to the predicted value of that sample. **(D–F)** Visualization of the prognostic model in the TCGA cohort, including **(D)** the distribution of patient risk scores, **(E)** the scatter plot of survival time and survival status, and **(F)** the expression heatmap of the 7 core hub genes stratified by risk groups.

Furthermore, univariate Cox proportional hazards regression analysis identified specific core hub genes (such as CHEK1, CCNA2, and PTK2B) as independent prognostic indicators for LUAD (Figure 5I). Multivariate Cox regression analysis confirmed that the prognostic risk model itself acted as a prognostic factor independent of other clinical characteristics (Figure 6A). Similarly, we conducted independent prognostic analyses for the model within the three GSE validation datasets; all results supported the model as an independent prognostic factor, with detailed results presented in Supplementary Figures S2A–C.

To enhance model interpretability, SHAP analysis was employed to quantify the contribution of each core hub gene to the risk predictions. The SHAP summary plots highlighted PTK2B, CCNA2, and SERPINE1 as the top three driving features of the model (Figures 6B,C). Finally, we visually mapped the distribution of patient risk scores, the corresponding survival statuses, and the expression patterns of the seven core genes (Figures 6D–F).

### 3.8 CCK8 and EdU

**FIGURE 7:**
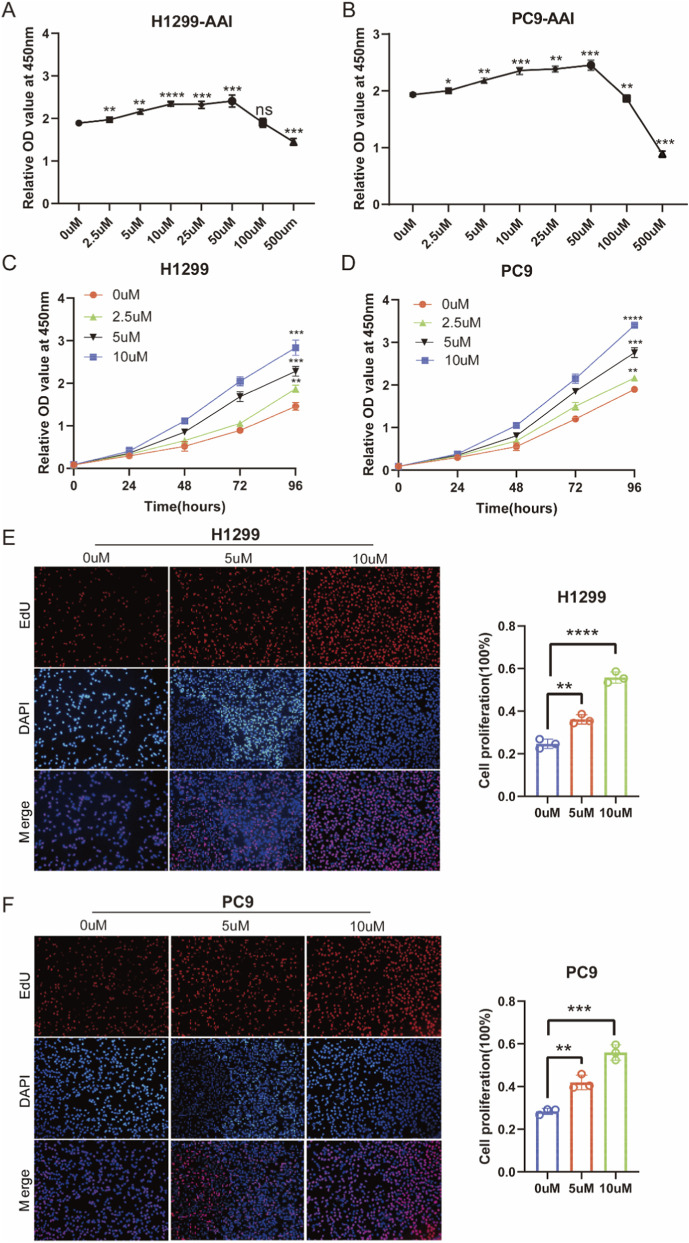
Cell proliferation assay. **(A)** Investigation of AAI Proliferative Concentration in the NCI-H1299 Cell Line. **(B)** Investigation of AAI Proliferative Concentration in the PC9 Cell Line. **(C)** Cell proliferation of NCI-H1299 over time at an AAI concentration of 2.5 μM, 5 μM, or 10 μM. **(D)** Cell proliferation of PC9 over time at an AAI concentration of 2.5 μM, 5 μM, or 10 μM. **(E)** EdU assay results of the NCI-H1299 cell line under 5 μM or 10 μM AAI treatment. **(F)** EdU assay results of the PC9 cell line under 5 μM or 10 μM AAI treatment. (*p < 0.05, **p < 0.01, ***p < 0.001, ****p < 0.0001).

We first evaluated the impact of AAI on LUAD cell viability across a broad concentration range (0–500 μM). The CCK-8 results showed that while high concentrations (>100 μM) inhibited growth, low micromolar doses significantly promoted the proliferation of both NCI-H1299 and PC9 cells, with the peak effect observed at 50 μM. For subsequent functional characterization, we focused on the physiologically relevant doses of 5 μM and 10 μM. Time-course CCK-8 assays revealed that AAI treatment accelerated cell growth in a dose-dependent manner over 96 h compared to the control group (P < 0.001; Figures 7A–D). Consistently, the EdU incorporation assay confirmed that 24-h exposure to 5 μM and 10 μM AAI significantly increased the proportion of proliferating cells in both cell lines, further validating the potent pro-proliferative effect of AAI at low doses (P < 0.01; Figures 7E,F).

### 3.9 Cell scratch and transwell assays

**FIGURE 8:**
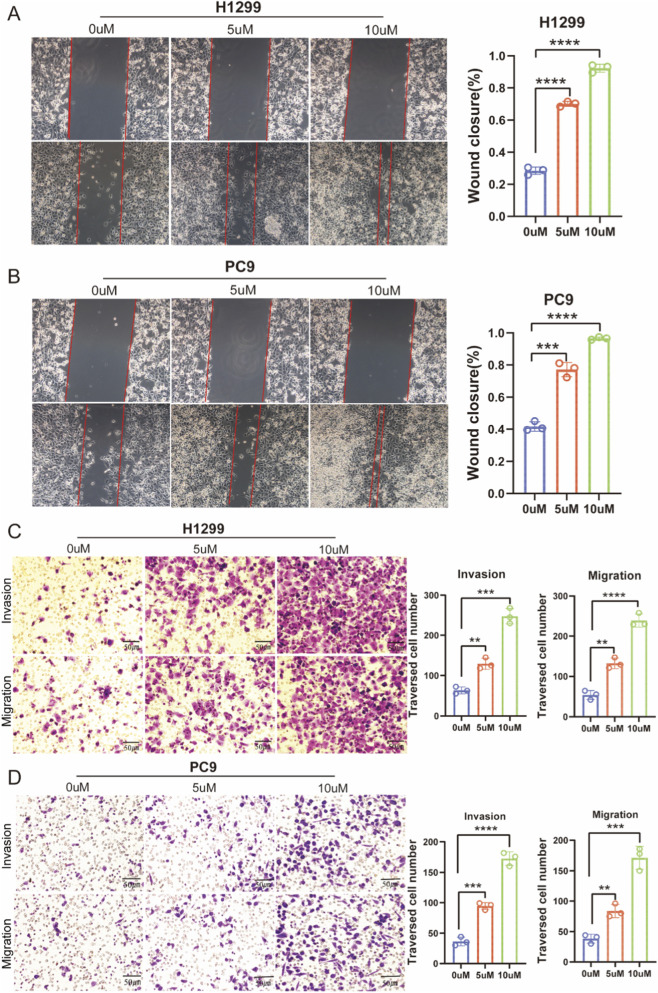
Cell proliferation and migration experiments. **(A)** The wound healing assay reflects the difference in migration speed of NCI-H1299 cells treated with 5 μM or 10 μM AAI compared to the control group. **(B)** The wound healing assay reflects the difference in migration speed of PC9 cells treated with 5 μM or 10 μM AAI compared to the control group. **(C)** In the Transwell assay, NCI-H1299 cells treated with 5 μM or 10 μM AAI showed significantly higher migration and invasion compared to the control group. **(D)** Transwell assay showed that PC9 cells treated with 5 μM or 10 μM AAI had significantly higher migration and invasion compared to the control group. (*p < 0.05, **p < 0.01, ***p < 0.001, ****p < 0.0001).

To investigate the influence of AAI on the aggressive behavior of LUAD cells, wound healing and Transwell assays were performed. The wound healing assay demonstrated that AAI-treated groups (5 μM and 10 μM) exhibited significantly higher wound closure rates at 30 h than the untreated control. Quantification showed that this migratory advantage increased with higher AAI concentrations (P < 0.0001; Figures 8A,B). Similarly, Transwell migration and invasion assays revealed that AAI exposure at 5 μM and 10 μM led to a dose-dependent increase in the number of traversed cells. These findings indicate that low-dose AAI significantly boosts the motility and invasiveness of LUAD cells *in vitro* (Figures 8C,D).

### 3.10 RT-qPCR

**FIGURE 9:**
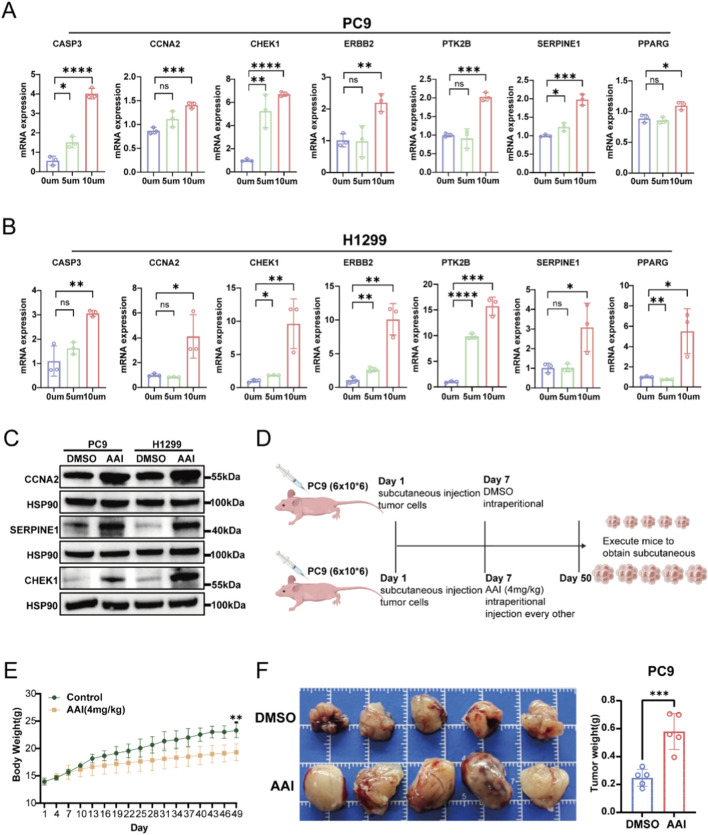
RT-qPCR, Western blot, and subcutaneous tumor formation experiments. **(A)** RT-qPCR results of the PC9 cell line treated with 0 μM, 5 μM, and 10 μM AAI. **(B)** RT-qPCR results of the H1299 cell line treated with 0 μM, 5 μM, and 10 μM AAI. **(C)** Western blot analysis confirming the protein expression levels of CCNA2, SERPINE1, and CHEK1 in PC9 and H1299 cell lines treated with DMSO or AAI (10 μM). **(D)** Subcutaneous tumor formation procedure in nude mice. **(E)** Weight change curve of nude mice. **(F)** Results and analysis of subcutaneous tumor formation in nude mice. (*p < 0.05, **p < 0.01, ***p < 0.001, ****p < 0.0001).

To validate the molecular response predicted by network toxicology at physiologically relevant concentrations, we performed RT-qPCR to measure the mRNA levels of the seven hub genes following treatment with 5 μM and 10 μM AAI. In both cell lines, AAI exposure triggered a robust and dose-dependent transcriptional upregulation of CASP3, CCNA2, CHEK1, ERBB2, PTK2B, SERPINE1, and PPARG. Specifically, in the PC9 cell line, while several genes including CCNA2, ERBB2, PTK2B, and PPARG showed non-significant (ns) changes at the lower dose of 5 μM, they all exhibited statistically significant upregulation upon exposure to 10 μM AAI (P < 0.05; Figure 9A). Similarly, in the NCI-H1299 cell line, although CASP3, CCNA2, and SERPINE1 did not show significant alterations at 5 μM (ns), a significant increase in the mRNA expression of all seven hub genes was observed at 10 μM (Figure 9B).

### 3.11 Western blot

To further validate the effect of AAI at the translational level, Western blot analysis was performed. After treating PC9 and NCI-H1299 cells with 10 μM AAI, the protein expression levels of CCNA2, SERPINE1, and CHEK1 were visibly upregulated compared to the DMSO control group (Figure 9C). These findings are very consistent with our RT-qPCR results, showing that AAI really does activate these key regulatory factors at the protein level.

### 3.12 *In vivo* tumor formation

To evaluate the tumor-promoting effects of AAI *in vivo*, a subcutaneous xenograft model was established in nude mice. Following the protocol illustrated in Figure 9D, mice were treated with 4 mg/kg AAI (Figure 9D). The results showed that AAI treatment led to a significant increase in both tumor volume and weight compared to the DMSO control group (P < 0.001). After AAI intervention, mice showed reduced body weight compared to the control group, while their food intake and mental status remained normal, and no death occurred before the scheduled sacrifice (Figure 9E). These *in vivo* findings complement our *in vitro* data, providing evidence that AAI exposure effectively promotes the macroscopic progression of LUAD tumors (Figure 9F).

## 4 Discussion

Aristolochic acids (AAs), a class of nitrophenanthrene carboxylic acids derived from Aristolochiaceae plants, have well-documented, severe nephrotoxic and carcinogenic effects (Arlt et al., 2002; Anger et al., 2020; Yang et al., 2025). Because early-stage lung adenocarcinoma (LUAD) often presents with non-specific respiratory symptoms like low-grade fever and chronic cough, patients may unknowingly consume AAI-containing herbal remedies for symptom relief. Using two toxicological prediction databases, ProTox 3.0 and ADMETlab 2.0, we confirmed the potent respiratory toxicity and carcinogenicity of AAI. While the chronic mutagenic effects of AAI-DNA adduct are well established, this study—by integrating network toxicology with multidimensional experimental validation—proposes that in preexisting LUAD, AAI may act as an “acute tumor promoter,” significantly accelerating the progression of malignant phenotypes.

In the *in vitro* experiments of this study, based on the CCK-8 screening results and with reference to previously reported toxicological models and epidemiological studies of AAI (Li Y. et al., 2023; Liu et al., 2022; Xie et al., 2024; Huang et al., 2025; Kwok and Chan, 2024; Lai et al., 2010), we selected experimental concentrations of 5 μM and 10 μM. These doses were utilized to simulate the cumulative tissue concentrations associated with chronic exposure, as well as the high localized concentrations potentially achieved via the respiratory route. The experimental results demonstrated that even at these sub-lethal concentrations, AAI could still significantly enhance the proliferation, migration, and invasion capabilities of LUAD cells within 24–48 h. For the *in vivo* experiments, a dose of 4 mg/kg was selected based on established protocols from the comprehensive literature (Sun et al., 2023; Liu et al., 2021; Huang et al., 2025; Kwok and Chan, 2024).

The traditional mechanism of AAI carcinogenesis primarily involves its metabolites forming DNA adducts (dA-AAI), which subsequently induce chronic A: T to T: A mutations in genes such as TP53 (Li P. L. et al., 2023; Bárta et al., 2021; Stiborová et al., 2017). However, the rapid phenotypic worsening we observed *in vitro* (within 24–48 h) cannot be fully explained by a slow, mutation-driven process. Instead, our data suggest that AAI interferes with pre-existing oncogenic transcriptional networks. According to our network enrichment analysis, the affected targets are significantly enriched in the ERK signaling pathway, focal adhesions, and chemical carcinogenesis pathways, providing a molecular basis for the malignant transformation induced by AAI (Eke and Cordes, 2015; Ullah et al., 2022).

Specifically, AAI accelerates LUAD progression through a dual axis of proliferation and invasion. Along the cell proliferation axis, the synergistic upregulation of ERBB2, CCNA2, and CHEK1 is critical. As a classic receptor tyrosine kinase, ERBB2 activation can trigger the MAPK/ERK and PI3K/AKT signaling pathways (Fanucci et al., 2024; Gao et al., 2021). Simultaneously, CCNA2 and CHEK1 serve as key regulators of the cell cycle and DNA damage repair. Their upregulation, which we confirmed at both the mRNA and protein levels, may allow tumor cells to bypass normal cell cycle arrest (Zhao et al., 2025; He et al., 2024). This process directly leads to the significant acceleration in proliferation observed in our study and further demonstrates that AAI promotes tumor growth by activating these targets (Qiu et al., 2018).

Along with the invasion and migration axis, the activation of SERPINE1 and PTK2B serves as a core driver. PTK2B plays a critical role in regulating cell motility (Wu et al., 2024), while extensive literature indicates that SERPINE1 promotes cancer progression, drives lung cancer invasion and metastasis, and correlates with a poor prognosis (Kong et al., 2021; Chai et al., 2024; Yuan et al., 2022). Although public TCGA data show a baseline downregulation of SERPINE1 in LUAD tissues, its expression may be stage-dependent. Our analysis of independent GEO datasets (GSE31210, GSE43458, and GSE81089) revealed that SERPINE1 is significantly upregulated in GSE81089, which included many advanced-stage LUAD patients. Furthermore, our *in vitro* experiments clearly demonstrate that AAI exposure significantly upregulates SERPINE1 at both the mRNA and protein levels. This upregulation directly correlates with the enhanced cell invasiveness observed in our study. These findings suggest that AAI can force the activation of this critical invasion gene, indirectly reflecting that AAI exposure leads to a poor prognosis for LUAD patients. The upregulation of CASP3 and PPARG in our study may be closely tied to cellular stress and metabolic changes under AAI exposure. Although PPARG is generally regarded as a tumor suppressor, recent studies indicate that it can also support cancer progression and metastasis by regulating lipid metabolism genes. Therefore, the increased expression of PPARG after AAI treatment might represent a specific metabolic response of LUAD cells to drug-induced stress (Li et al., 2026; Tang et al., 2023). This complex mechanism requires deeper study in the future (Samarasekera et al., 2025; Xiong et al., 2025).

In this study, a prognostic risk model was established based on the seven core hub genes. Multivariate Cox regression analysis confirmed that the risk score can serve as an independent predictor of poor prognosis, operating independently of clinical characteristics such as age, gender, and tumor stage. Furthermore, by introducing three large, independent external cohorts (GSE31210, GSE68465, and GSE81089) for cross-validation, we showed that this model is both versatile and stable. As multigene risk models increasingly guide precision oncology (Kattan et al., 2016), ensuring their interpretability has become paramount. To dismantle the ‘black box’ nature of complex predictive models, we employed SHAP analysis to enhance the interpretability of the risk model (Lundberg et al., 2018). This approach successfully combines global feature importance with individual sample risk weights, clearly identifying PTK2B, CCNA2, and SERPINE1 as the top three core elements driving prognostic risk, thereby imparting clear clinical significance to our computational predictions.

Admittedly, this study has certain limitations. We have primarily demonstrated a strong correlation between AAI exposure and the activation of oncogenic networks; future gene-specific knockdown or overexpression experiments are still needed to establish absolute causal relationships for individual genes. Furthermore, the rapid phenotypic alterations observed *in vitro* might partly reflect a generalized cellular stress response. Like other severe external stressors (Andalib et al., 2024), acute AAI exposure could trigger adaptive survival and inflammatory cascades in pre-existing LUAD cells. Future research should investigate the crosstalk between these toxicant-induced stress responses and our identified core gene networks. While molecular docking provides theoretical insights into potential AAI-protein interactions, it remains purely an exploratory computational tool. Due to the inherent limitations of *in silico* predictions, subsequent biophysical validations are indispensable to confirm actual biological binding (Sadybekov and Katritch, 2023; Sliwoski et al., 2014). Finally, although our *in vivo* model confirmed the macroscopic acceleration of tumor growth, the pathological characterization of the collected tissues still needs further study. In addition, this study has limitations in toxicity assessment. In our future AAI toxicity studies, we will comprehensively evaluate organ toxicity by including histopathological analysis of liver and kidney sections and serum biomarkers.

## 5 Conclusion

In summary, our study provides multidimensional evidence suggesting that, beyond its established role as a chronic mutagen, AAI may act as a potent acute tumor promoter in preexisting LUAD. This accelerated malignant progression is closely associated with the rapid transcriptional upregulation of a core oncogenic network, which predominantly drives tumor proliferation and invasion *in vitro* and promotes macroscopic tumor growth *in vivo*. These findings expand our understanding of AAI-induced toxicity and highlight the severe clinical risks of AAI exposure for patients with preexisting lung malignancies, underscoring the urgent need for stricter pharmacovigilance and exposure restrictions in this vulnerable population.

## Data Availability

Publicly available datasets were analyzed in this study. This data can be found here: https://www.cancer.gov/ccg/research/genome-sequencing/tcgaformat.
