# Supplementary material for: Aristolochic acid I accelerates lung adenocarcinoma progression coupled with the upregulation of core oncogenic networks: an integrated network toxicology and experimental study
Source: Front Pharmacol. 2026 Jul 15;17:1860873. doi: 10.3389/fphar.2026.1860873 (PMC13415939; doi:10.3389/fphar.2026.1860873)
Supplement: Supplementary file 1 [file Supplementaryfile1.docx]

**Aristolochic Acid I Accelerates Lung Adenocarcinoma Progression Coupled with the Upregulation of Core Oncogenic Networks: An Integrated Network Toxicology and Experimental Study**

**Supplementary Information(SI)**

| **Gene** | **Primers(5‘-->3’)** |
| --- | --- |
| CHEK1 | Forward CGTGACGCCCTCAAGTTTTG  Reverse TCCATAGGCACCTTCTCCCA |
| CCNA2 | Forward GCACTGGTGGTCTGTGTTCT  Reverse TGGATGCCAGTCTTACTCATAGC |
| PPARG | Forward AGCCTGCATTTCTGCATTCTG  Reverse TGGCATCTCTGTGTCAACCA |
| CASP3 | Forward TCCTAGCGGATGGGTGCTAT  Reverse CTCACGGCCTGGGATTTCAA |
| PTK2B | Forward GGAGCGTGGATGATCTCCTG  Reverse CTCACTTAGGGAGGTCACGG |
| SERPINE1 | Forward AGAGCGCTGTCAAGAAGACC  Reverse AGTTCTCAGAGGTGCCTTGC |
| ERBB2 | Forward GTTCCCGGATTTTTGTGGGC  Reverse CATGGGGAAGCAATCACCCT |
| B-ACTIN | Forward TCCCTGGAGAAGAGCTACGA  Reverse TGAAGGTAGTTTCGTGGATGC |

**A. Supplementary Table**

**Table. S1.** Primers for RT-qPCR detection of tumor cells

**B. Supplementary Figures**


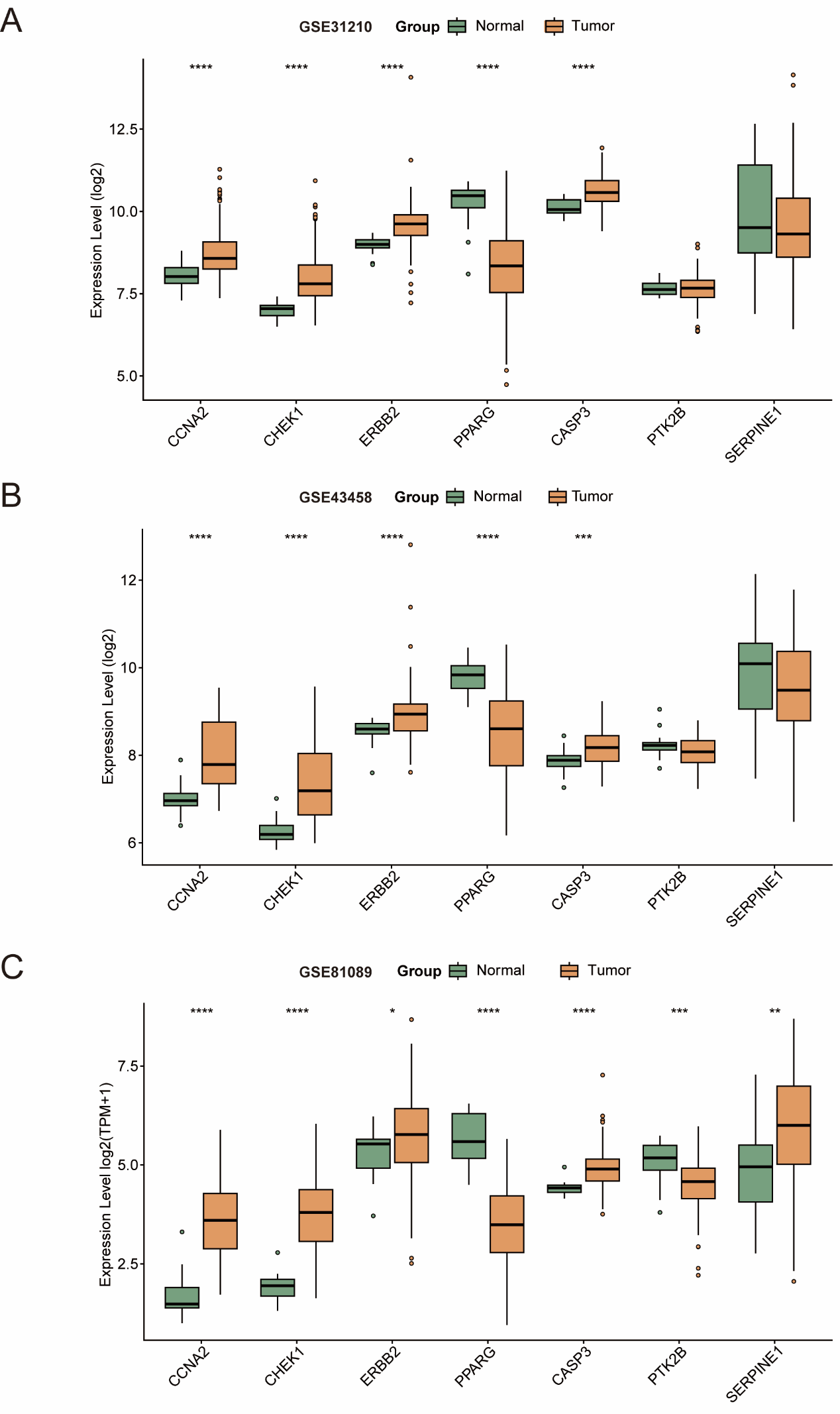


**Fig. S1. Expression of core genes in different groups in three GEO validation datasets.** **(A)** GSE31210. **(B)** GSE43458. **(C)** GSE81089*. (*: p<0.05, **: p<0.01, ***: p<0.001, ****: p<0.0001)*

*
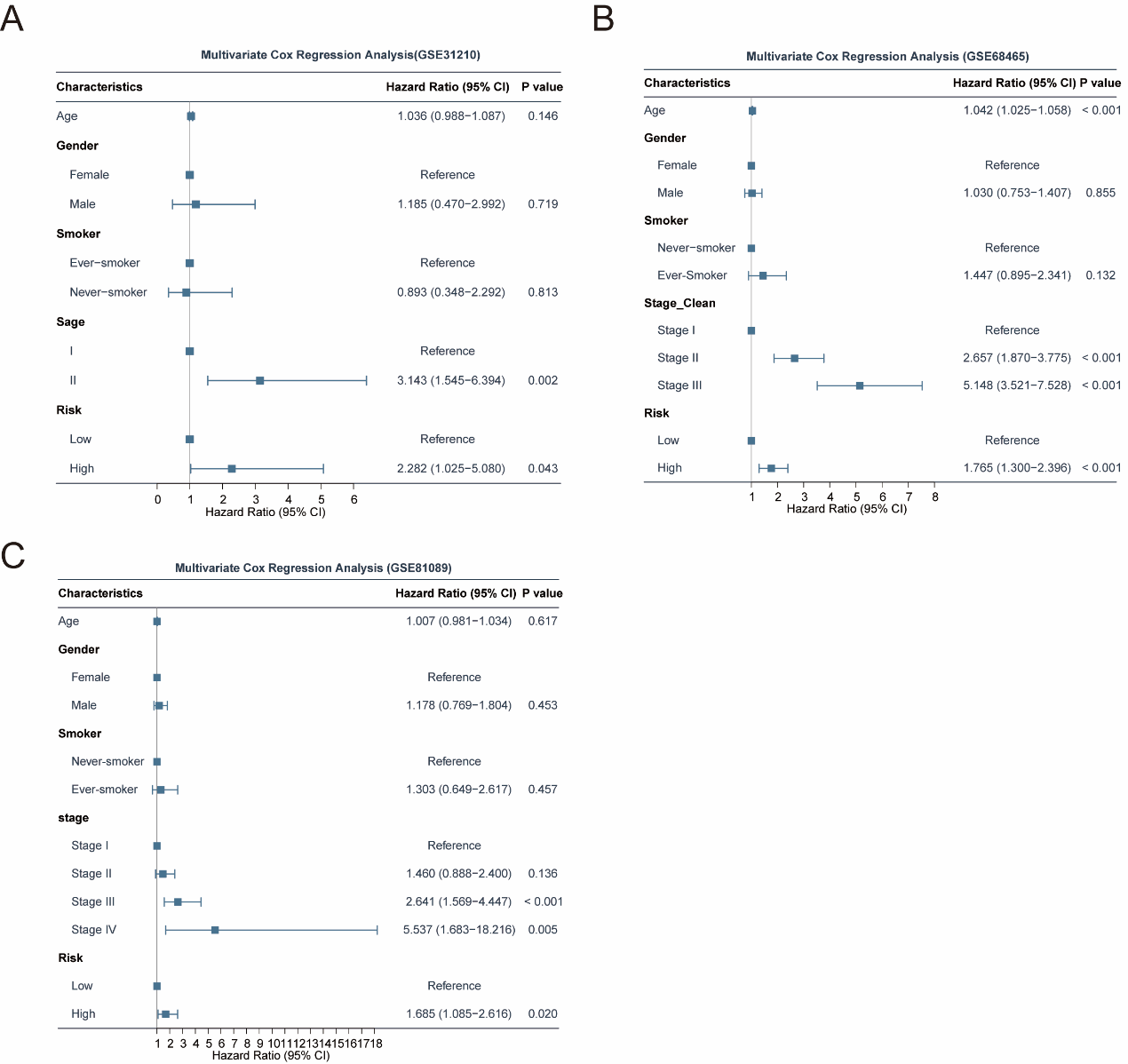
*

**Fig. S2. Multivariate Cox regression analysis (forest plot) incorporating clinical characteristics (Age, Gender, Smoker, Stage) and the Risk score. (A)** GSE31210. **(B)** GSE68465. **(C)** GSE81089.


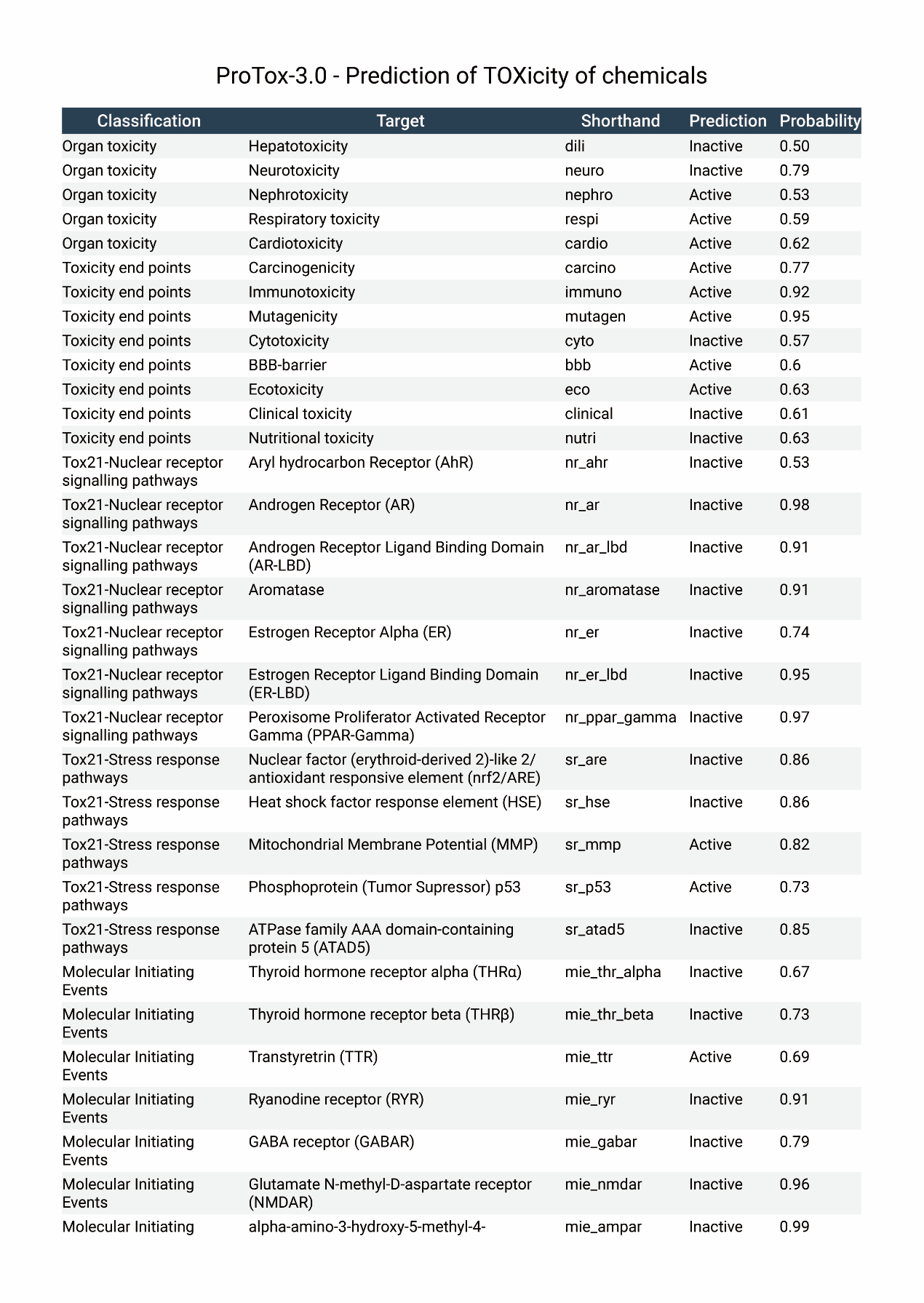


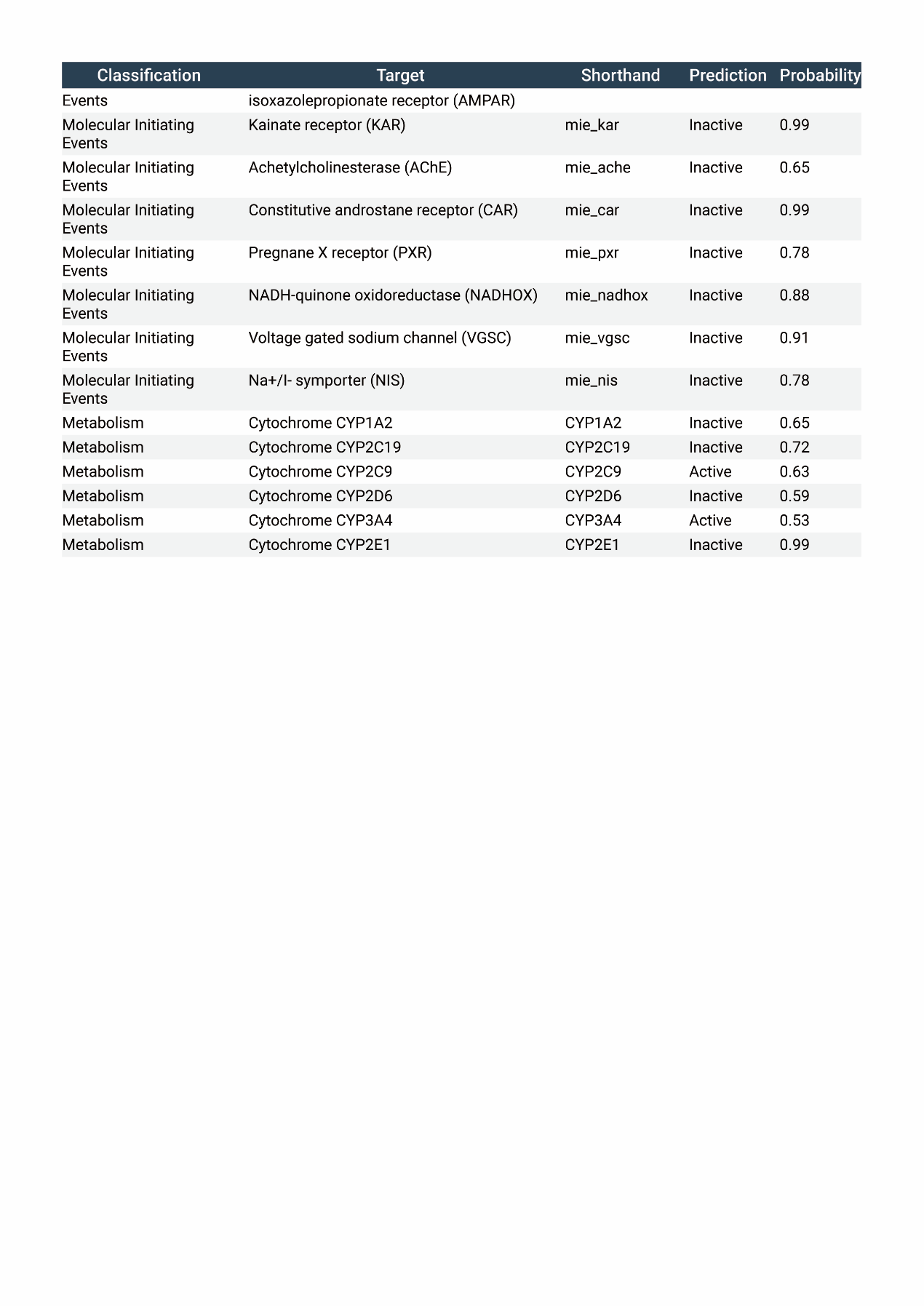


**Fig. S3.** Protox3.0 Toxicity Prediction for AAI.


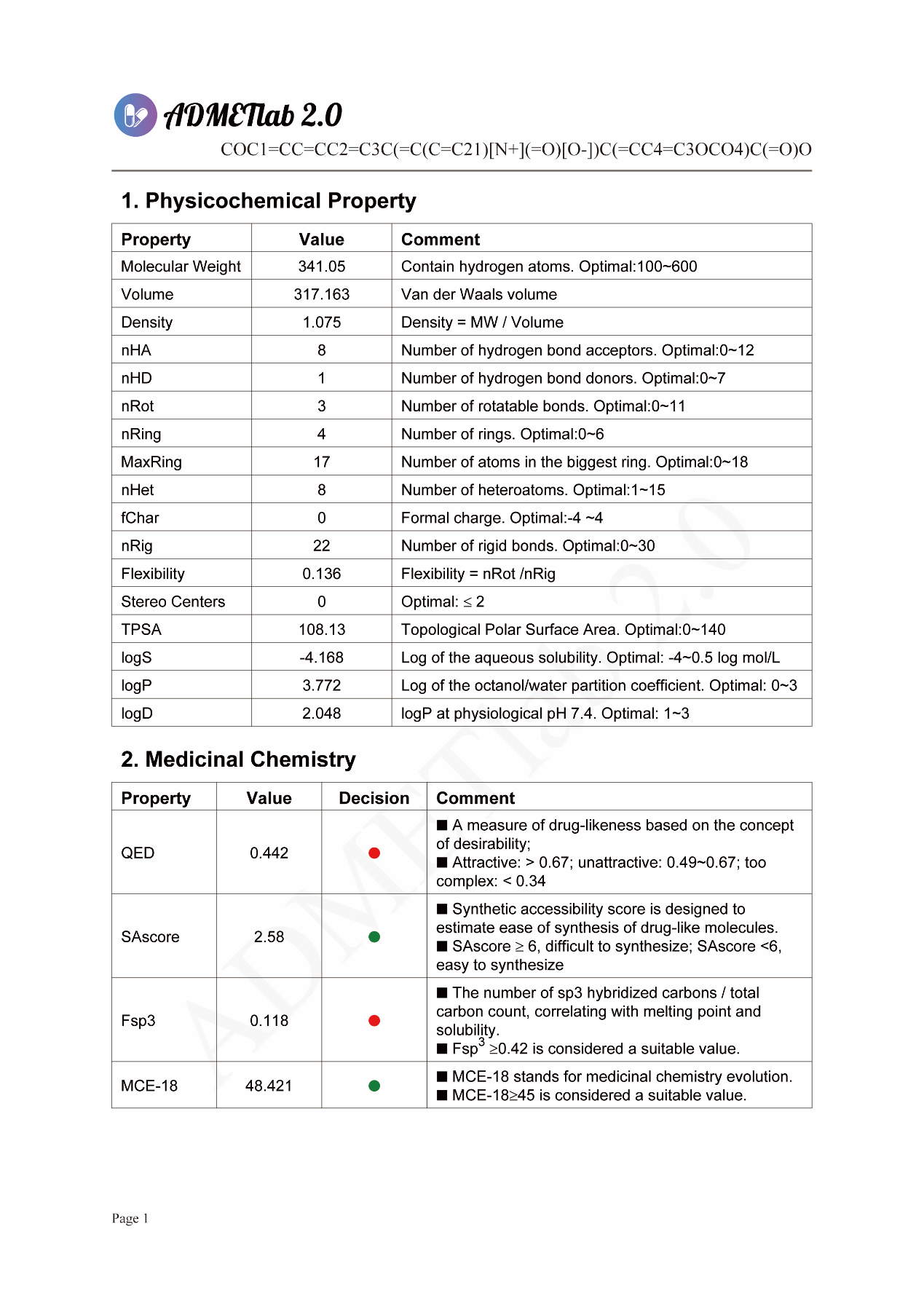


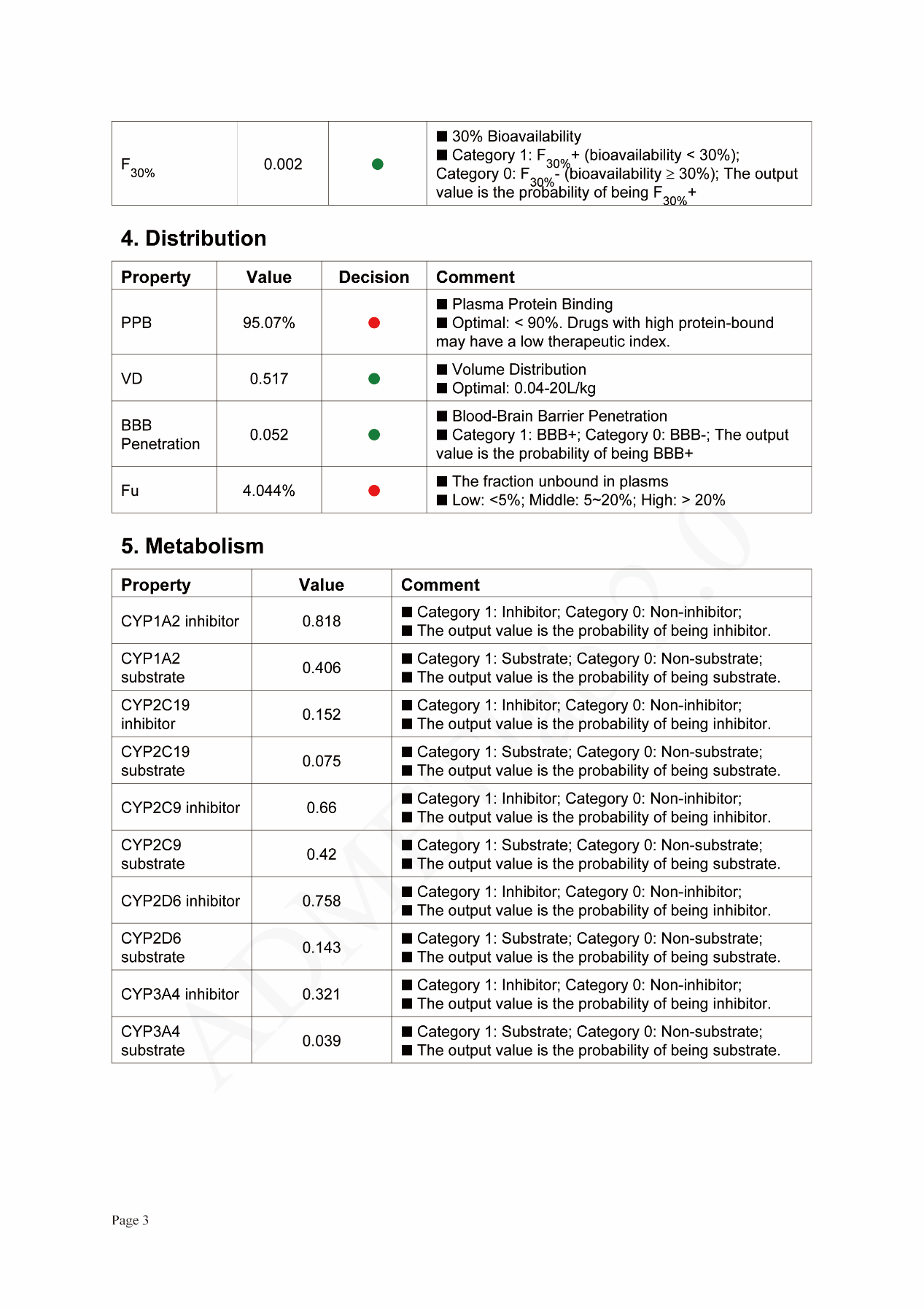

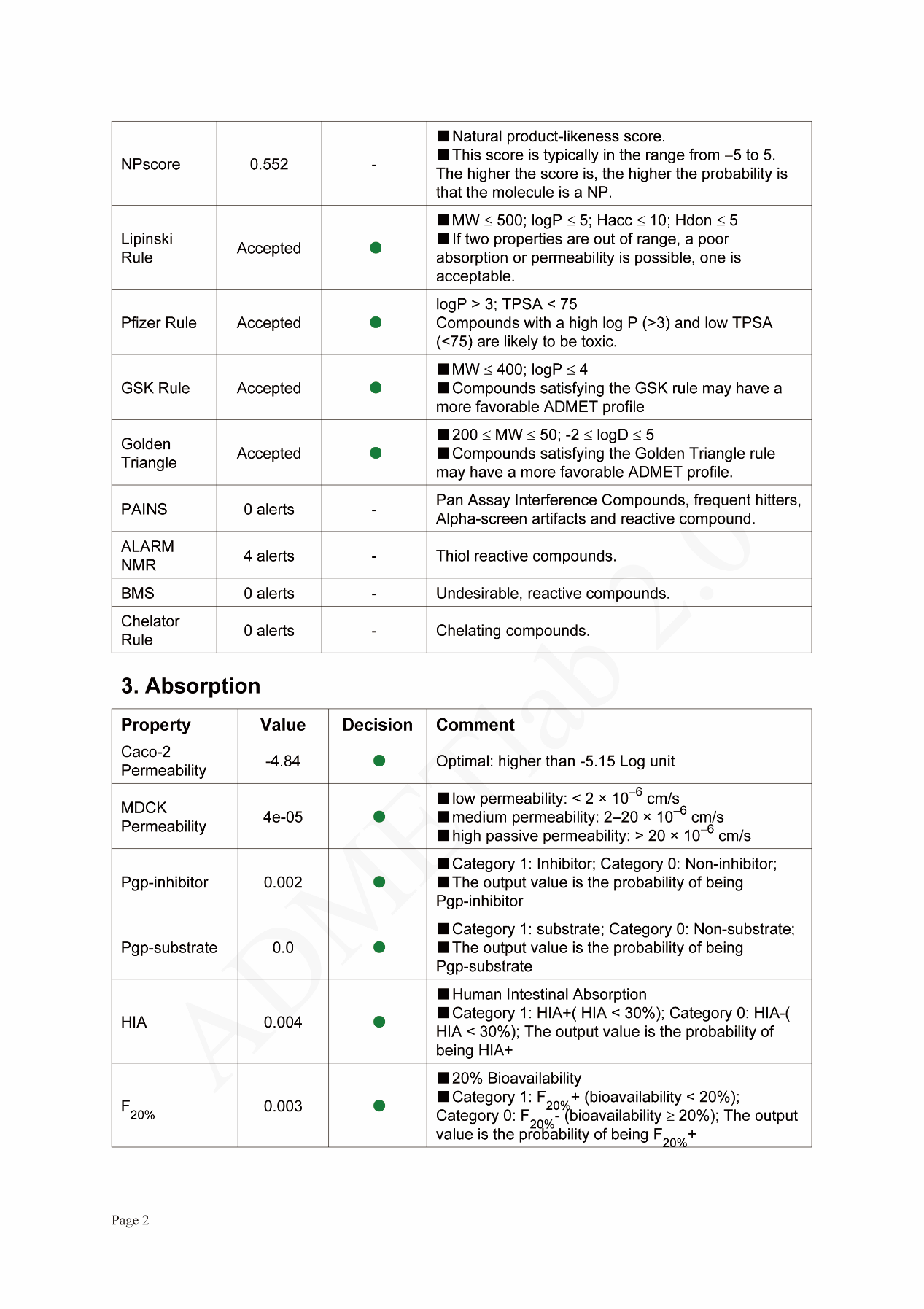


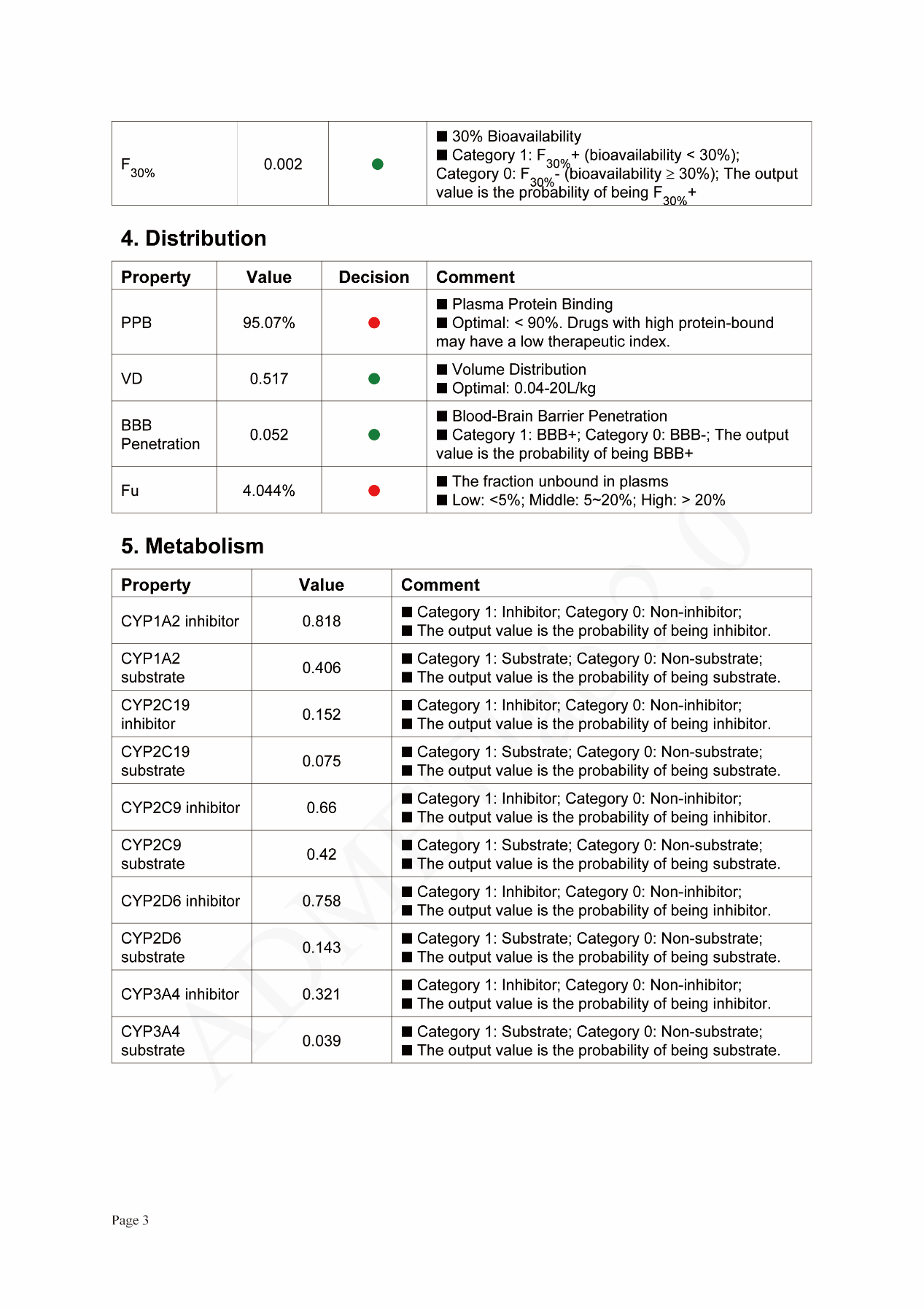


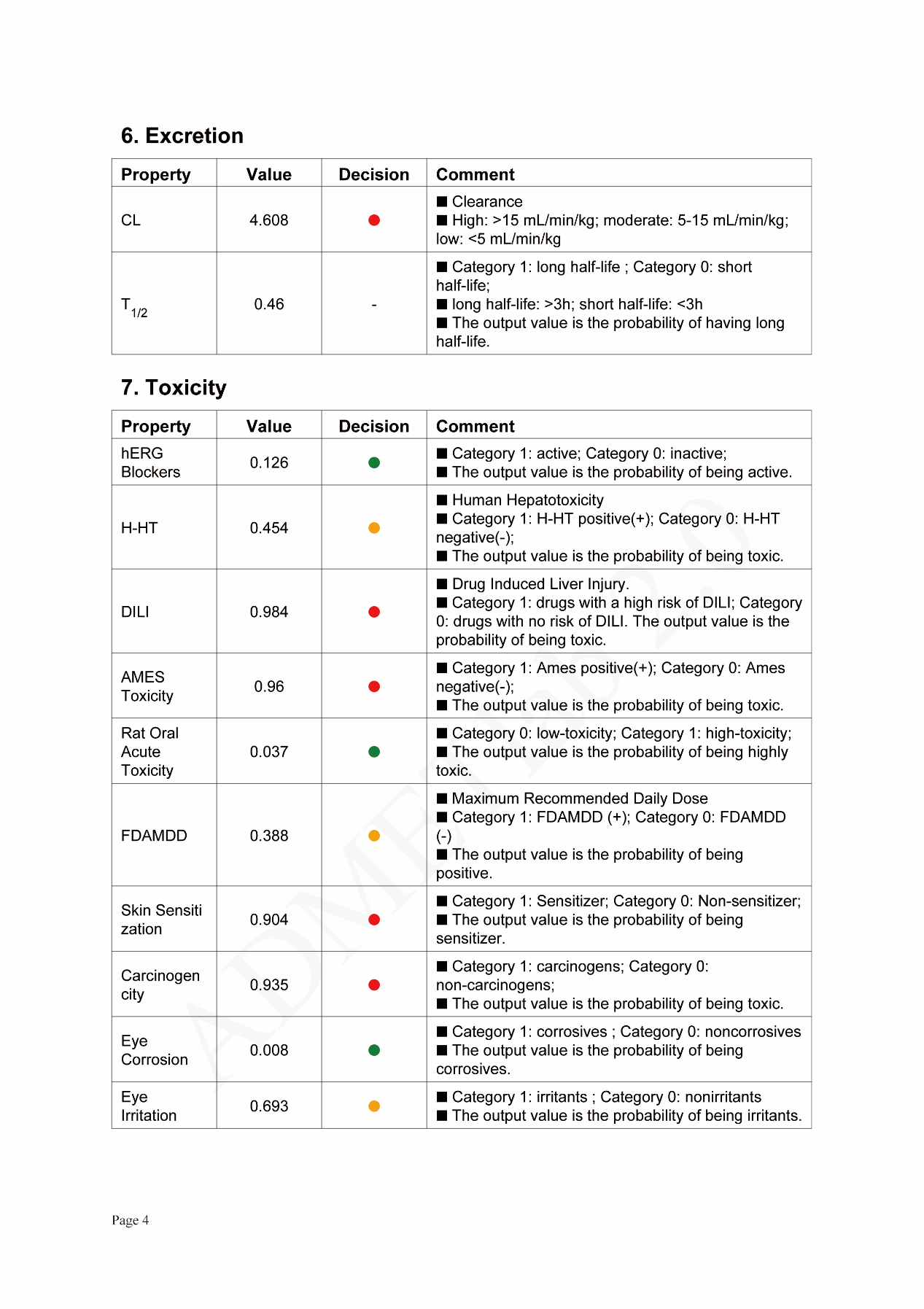


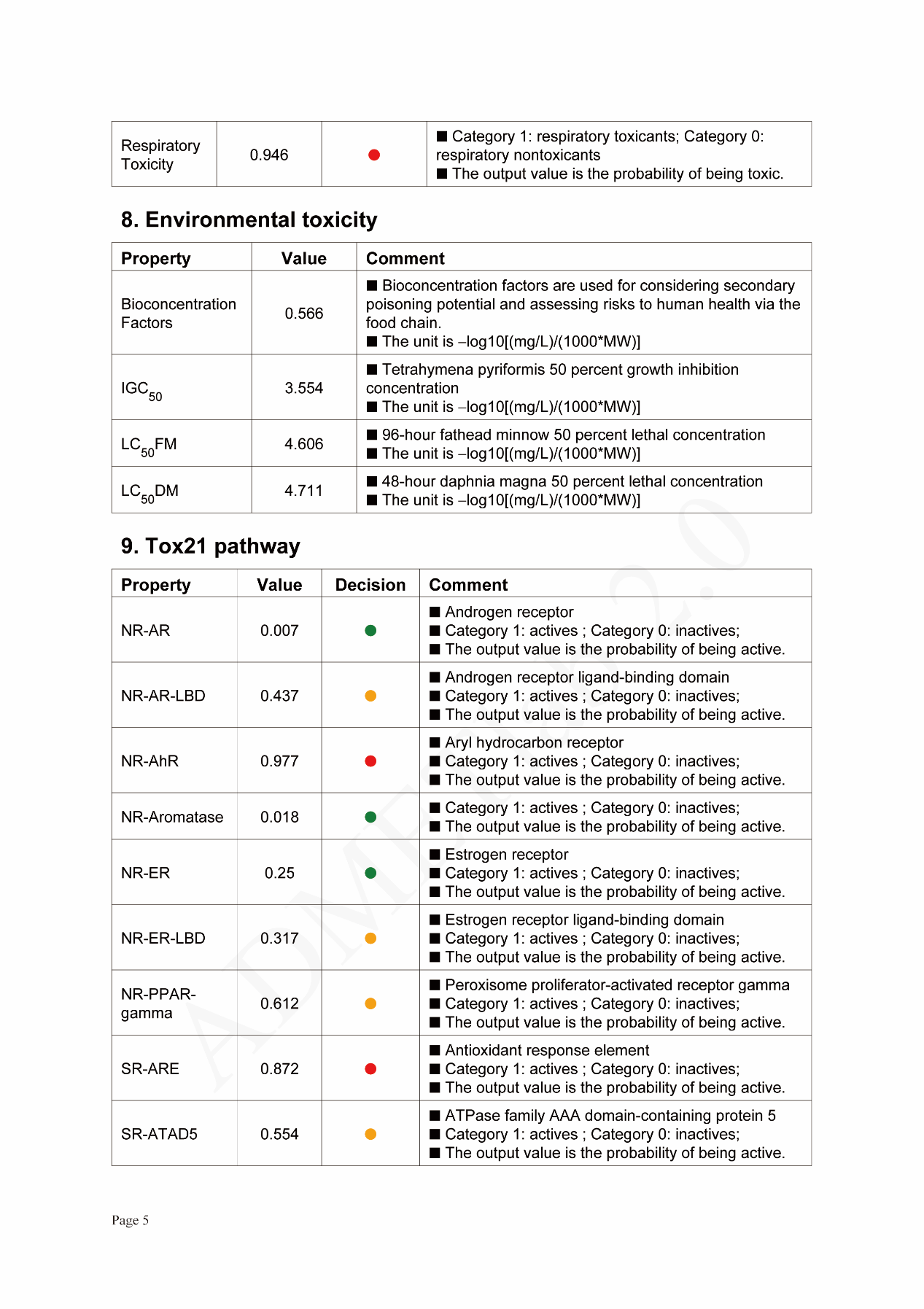


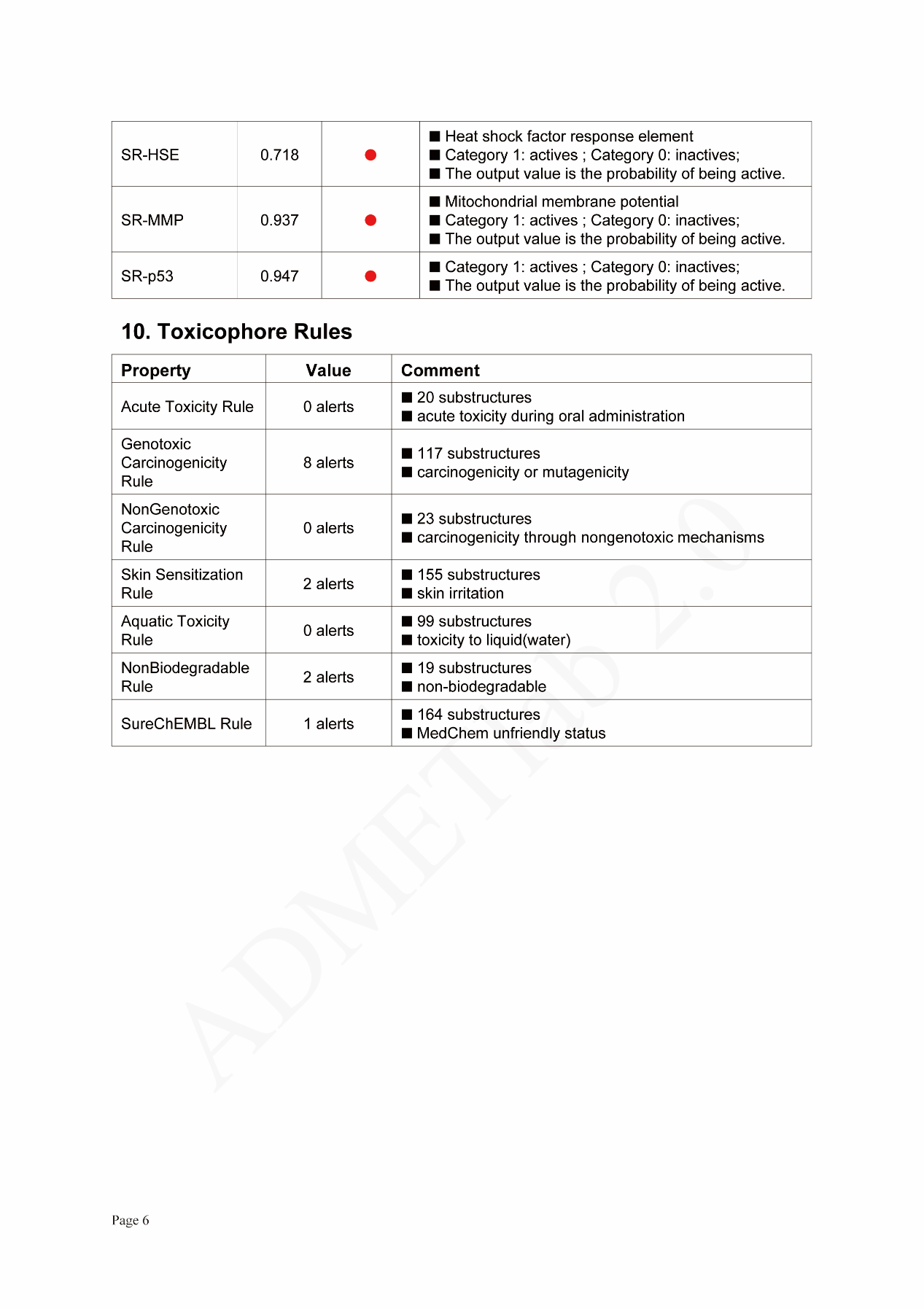


**Fig. S4.** ADMETab 2.0 Toxicity Prediction for AAI.
